# Prevalence of bovine trypanosomosis in Ethiopia: a meta-analysis

**DOI:** 10.1186/s13071-016-1404-x

**Published:** 2016-03-10

**Authors:** Samson Leta, Gezahegn Alemayehu, Zewdu Seyoum, Melkamu Bezie

**Affiliations:** Faculty of Veterinary Medicine, University of Gondar, P.O. Box: 196, Gondar, Ethiopia; College of Veterinary Medicine, Samara University, Samara, Ethiopia

**Keywords:** Bovine trypanosomosis, Ethiopia, Meta-analysis, Prevalence

## Abstract

**Background:**

Trypanosomosis is a haemoprotozoan disease, mostly transmitted by the tsetse fly (*Glossina* spp.), it causes severe disease in humans and animals in Sub-Saharan Africa (SSA). The disease results in loss of livestock and agricultural productivity with severe socio-economic impacts. In Ethiopia, bovine trypanosomosis is widely distributed in western and south-western parts of the country. It is estimated that some 10 to 14 million heads of cattle in Ethiopia are exposed to the risk of trypanosomosis.

**Methods:**

This study describes the prevalence of bovine trypanosomosis in Ethiopia through a meta-analysis. A comprehensive search was conducted on PubMed and non-PubMed indexed articles were also incorporated based on expert suggestion. Eligible studies were selected by using inclusion and exclusion criteria. Pooled prevalence was estimated by random effect model. Publication bias and the variation in prevalence estimates attributed to heterogeneity were also assessed.

**Results:**

Twenty-four studies with relevant prevalence data were identified and included in the analysis. The apparent prevalence of bovine trypanosomosis varied from 1.38 to 17.15 %. The pooled estimate of bovine trypanosomosis prevalence across studies for the entire period was 8.12 % (95 % CI: 6.88; 9.35), ranging from 10.27 % (95 % CI: 7.34; 13.20) in the late 1990s and early 2000s, to 6.81 % (95 % CI: 5.00; 8.62) after 2010. Sub-analysis by region revealed wide variations in prevalence. The highest estimated regional prevalence was 13.30 % (95 % CI: 7.73; 18.88) in Benishangul Gumuz Regional state. A high degree of heterogeneity was observed in most pooled estimates and even after sub-group analysis. The visual inspection of the funnel plot and the Egger’s regression asymmetry coefficient [*b* = 2.18] (95 % CI = −1.09, 5.46; *p* > 0.05) did not suggest the presence of publication bias. *T. congolense* and *T. vivax* were reported to be the predominant causative agents. From the total positive animals, 45.5 and 44.3 % of the infections are accounted to *T. congolense* and *T. vivax*, respectively.

**Conclusions:**

The meta-analysis showed a significant reduction in prevalence of bovine trypanosomosis in recent years, but the reduction is not to the lowest necessary level. Since *T. vivax* is reported to be one of the most important trypanosome species involved, efforts should also be made to control the mechanical transmission by biting flies.

**Electronic supplementary material:**

The online version of this article (doi:10.1186/s13071-016-1404-x) contains supplementary material, which is available to authorized users.

## Background

According to the Food and Agricultural Organization of the United Nations, trypanosomosis is probably the only disease which has a significant effect on the settlement and socio-economic development of a major part of SSA. Of the approximately 7–10 million km^2^ of land that are infested by tsetse fly only 20 million cattle are reared. If it is free from tsetse and trypanososmosis, this land could support more than 140 million heads of cattle and increase meat production by 1.5 million tons [1]. Bovine trypanosomosis causes about 3 million deaths every year and approximately 35 million doses of trypanocidal drugs are being administered every year to enable livestock to survive in tsetse-infested areas. While the economic losses in cattle production alone is up to US$1.2 billion, the indirect impact engendered by the disease on the agriculture-livestock production is estimated to be about US$4.5 billion a year [2]. The overall negative impact of trypanosomosis extends to the access and availability of cultivable areas, changes in land use and exploitation of natural resources, restriction of opportunities for agricultural diversification and intensification.

Trypanosomosis directly affects the milk and meat productivity of animals, reduces birth rates, increases abortion as well as mortality rates; all of these reduce the herd size and herd composition. The indirect impact of the disease mostly lies on crop production through the availability and cost of animals that provide traction power [3]. Trypanosomosis reduces work efficiency of oxen and discourages the introduction of drought animals in to crop farming [4]. Shaw et al. [5] discussed the economic benefits from intervening against bovine trypanosomosis. These authors reported significant benefits especially for Ethiopia, because of its very high livestock densities and the importance of animal traction. The estimated maximum benefit per square kilometer of tsetse infested area over a 20 year period is US$10,000. Consequently, the total maximum benefits from dealing with bovine trypanosomosis in Ethiopia could be as much as US$1 billion over a 20 year period.

In Ethiopia, significantly large numbers of works have been conducted to determine the prevalence of bovine trypanosomosis. However, the studies were limited in spatial scope and the results significantly vary between the studies. In this study, we used previously conducted studies to determine the overall prevalence of bovine trypanosomosis using meta-analysis. Disease control and prevention should be based on transparent and evidence-based planning. Unfortunately, the information in the literature is often inconclusive and could be conflicting. Meta-analysis has been defined as: “The statistical analysis of a large collection of analysis results from individual studies for the purpose of integrating the findings” [6]. It is a formal process for combining results from a number of studies that is being used increasingly in human medicine and, to a more limited extent, in veterinary medicine. A more complete description of meta-analyses can be found in  [7, 8].

Therefore, the objective of this study was to estimate the overall prevalence of bovine trypanosomosis in Ethiopia based on data from a number of scientific studies.

## Methods

The study was conducted based on the guideline of the Preferred Reporting Items for Systematic Reviews and Meta Analyses the (PRISMA) group checklist [9]. The checklist was used to ensure inclusion of relevant information (Additional file 1). The outcome of interest was the proportions of cattle infected with different species of trypanosomes.

### Study area

The study was conducted in Ethiopia; a country situated in the horn of Africa located between 3° 00′–15^0^ 00′ N latitude and 32^0^ 30′–48^0^ 00′ E longitude. It covers a land area of 1.04 million km^2^. With a population of 94.10 million, Ethiopia is the second most populous nation in Africa following Nigeria [10]. Ethiopia is suitable for agricultural production and it is also a home for an estimated 54, 25.5 and 24 million heads of cattle, sheep and goat, respectively [11].

The country has a diverse topography, which forms the basis for several agro-climatic zones. The area above 2300 m above sea level (m.a.s.l.) is considered highland, which is surrounded by a temperate transition zone between 1500 and 2300 m.a.s.l. Areas having an altitude below 1500 m.a.s.l. are classified as lowlands.

### Literature search and eligibility criteria

Comprehensive literature searches were conducted on PubMed (covering all dates from the creation of each database up to November 29, 2015). Ethiopian experts in the field were contacted to suggest other potential articles which were published in non-PubMed indexed journals. The following MeSH terms were used in electronic database search: “Trypanosomiasis”, “Trypanosomosis”, “Bovine trypanosomosis”, “Bovine trypanosomiasis”, “Prevalence of bovine trypanosomosis”, “Prevalence of bovine trypanosomiasis” and “Ethiopia”. Systematic reviews of available literature were performed to identify relevant publications about the prevalence of bovine trypanosomosis in Ethiopia. First, titles and abstracts were assessed, and respective papers were examined in detail. To be eligible, the following inclusion criteria were used: a study had to be (i) published in a  reputable journal, (ii) written in English, (iii) cross-sectional study and (iv) conducted in Ethiopia (v) number of infected animals, size of study population and test method available (vi) published as of 2000. Mendeley version 1.15.2 was used to catalogue the initial literature search results and to manage citations.

### Data extraction

From eligible studies, the following data were extracted: the first author, year of publication, year of study, study design, location, sampling design, sample size, test methods and prevalence. Prevalence of trypanosomosis was defined as the frequency of cases by *T. congolense*, *T. vivax* and/or *T. brucei* infection in a given population at a given period of time. The study level estimates and confidence intervals were derived from the extracted data.

### Data synthesis and analysis

Data analysis was carried out in different steps. First, mean prevalence was calculated, using the sum of the numbers of cases in all studies considered, divided by the sum of the number of cattle sampled. The 95 % confidence interval (95 % CI) was computed using exact binomial method. If the study did not report the year of data collection, the preceding year of the year of publication was considered. The pooled prevalence estimates for bovine trypanosomosis in the general population and their 95 % CI were calculated using the random-effects model meta-analysis [12].

Heterogeneity between studies was evaluated through the Cochran’s Q test (reported as *p* value) and inverse variance index (*I*^2^), which describes the percentage of observed total variation between studies that are due to heterogeneity rather than chance. The *I*^2^ values of 25, 50, and 75 % show low, moderate, and high degrees of heterogeneity, respectively. The *I*^2^ values 0 % indicate no observed heterogeneity. Q is the weighted of squares on a standardized scale. It is reported with a *p* value with low *p* values indicating the presence of heterogeneity [13].

Sub-group analyses were performed to determine the potential sources of heterogeneity among studies. The variables included in the sub-group analysis were: study area (region), sample size and year of the study.

The across study bias was first visually examined by a funnel plot, and then Egger’s regression asymmetry test was used to test the statistical significance of the bias [14]. The unbiased estimates were calculated using Duval and Tweedie non-parametric ‘fill and trim’ linear random method [15].

For each study, the prevalence with corresponding 95 % CI and the overall random-effects pooled estimate of all the studies were presented.

Data were analysed using Stata software version 12 (StataCorporation, College Station, USA) and Comprehensive Meta-Analysis software version 3.3.070 (Biostat, Englewood, USA). A map detailing the prevalences at study sites was created, using Quantum GIS software version 2.0.1 (Open Source Geospatial Foundation, Boston, USA).

## Result

### Literature search results

**Fig. 1:**
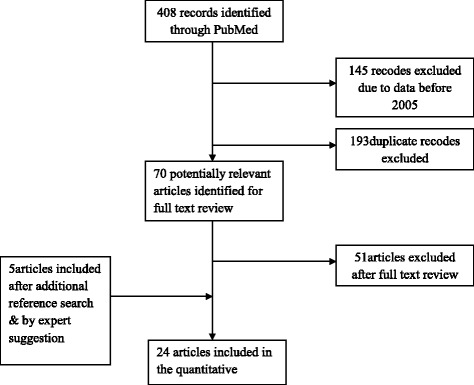
Flow diagram of the selection of eligible studies

Of 408 studies identified through electronic search and 5 studies suggested by the experts, 24 were considered for the meta-analysis [16–39]. Figure 1 the flow diagram of eligible study selection. These included a total of 27,719 cattle, with 2,274 cases. *T. congolense* and *T. vivax* were the predominant cause of the infection. From the total positive animals 45.5 and 44.3 % of them accounted to *T. congolense* and *T. vivax*, respectively and the remaining accounted to *T. brucei*.

### Characteristics of the studies

The studies were conducted between 1997 and 2014 in 6 regional states, namely Afar, Amhara, Beneshangul Gumuz, Oromia, South Nations Nationalities and Peoples Region (SNNPR) and Tigray. The sample size ranged from 250 to 7,079 cattle (mean: 1155; standard deviation [SD±]: 1525). In all the studies Haematocrit centrifugation technique (HCT) was used for the diagnosis of the infection. The apparent prevalence of bovine trypanosomosis varied from 1.38 to 17.15 % (mean: 8.28; standard deviation [SD±]: 3.64). Detailed characteristics of the studies included are presented in Table 1.

**Table 1:** A list of the studies included in the meta-analysis

Author (year)	Study year	Sample size	No. positive	Apparent prevalence
Sheferaw et al. 2015 [32]	2014	1838	133	7.24
Birhanu et al. 2015 [19]	2013	493	36	7.30
Lelisa et al. 2015 [28]	2014	405	22	5.43
Terefe et al. 2015 [36]	2014	409	25	6.11
Abera et al. 2014 [16]	2014	384	24	6.25
Biyazen et al. 2014 [21]	2014	384	11	2.86
Lelisa et al. 2014 [27]	2010	389	42	10.80
Tamiru et al. 2014 [35]	2013	436	6	1.38
Tafese et al. 2012 [34]	2011	386	33	8.55
Tesfaye et al. 2012 [37]	2009	1260	153	12.14
Fikru et al. 2012 [24]	2011	1524	81	5.31
Bishaw et al. 2012 [20]	2011	384	30	7.81
Bekele & Nasir, 2011 [18]	2011	384	33	8.60
Mekuria & Gadissa, 2011 [29]	2009	540	67	12.41
Dagnachew & Shibeshi, 2011 [23]	2009	368	33	8.97
Tadesse & Tsegaye, 2010 [33]	2009	250	11	4.40
Kebede & Animut, 2009 39	2008	3200	322	10.06
Miruk et al. 2008 31	2007	341	40	11.73
Mihret & Mamo, 2007 30	2005	3360	275	8.20
Sinshaw et al. 2006 [48]	2004	1509	92	6.10
Cherenet et al. 2004 22	2001	7079	501	7.08
Tewelde et al. 2004 38	2001	904	70	7.74
Kidanemariam et al. 2002 26	2001	1008	151	14.98
Afewerk et al. 2000 17	1997	484	83	17.15

### Geographical distribution of study sites

**Fig. 2:**
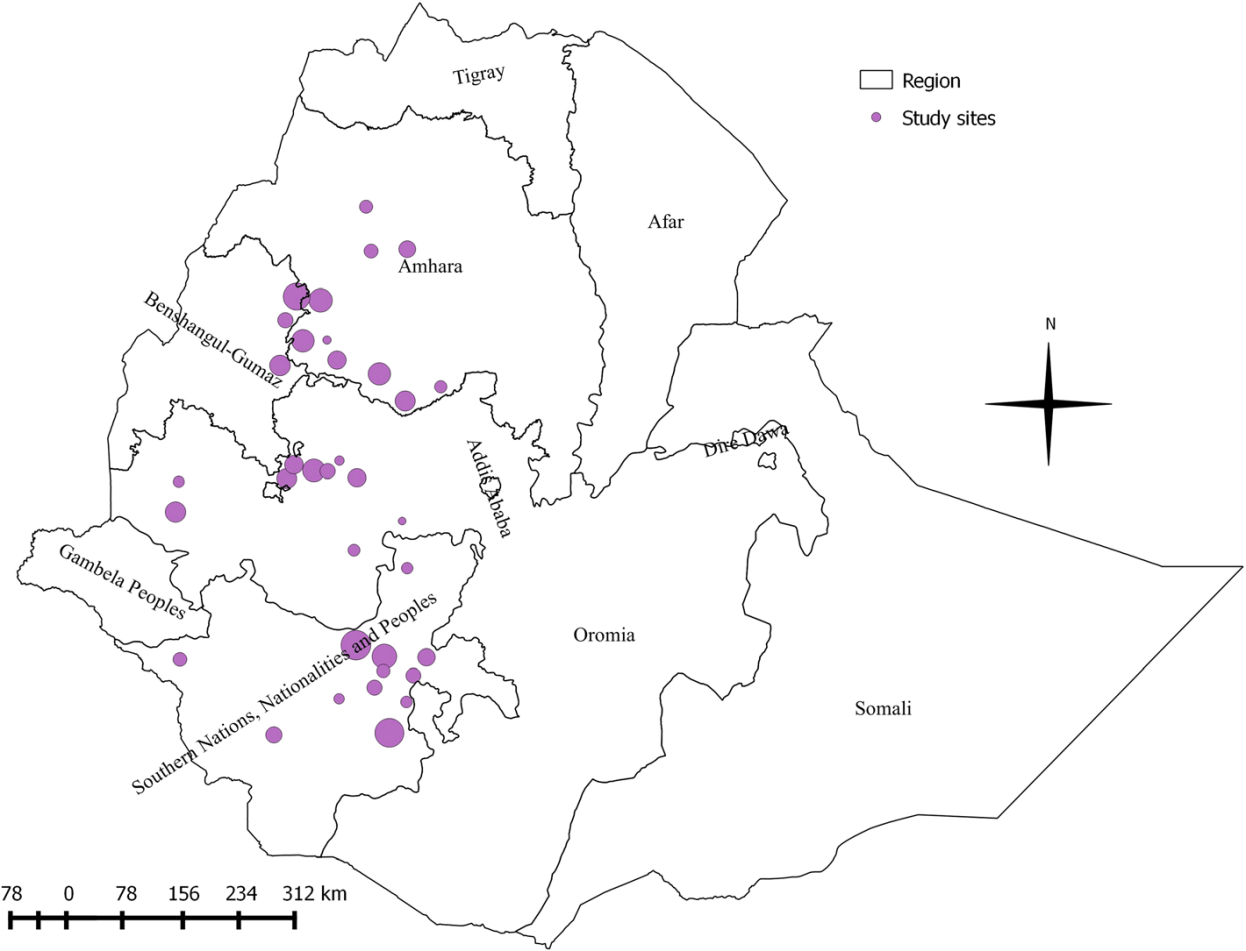
Observed spatial distribution of bovine trypanosomosis in Ethiopia

A total of 49 survey districts were identified from the 24 studies; however, five sites were not included into the map due to lack of unique location identifier. Spatial distribution and observed prevalence (size of the point is proportional to the prevalence) by location are depicted in Fig. 2. Most studies were performed in the western and south-western parts of the country. Some states, namely Amhara, Benishanguz Gumuz, Oromia and SNNPR contain a large number of survey locations, while other states, namely Afar and Tigray have only a few disease surveys published. The mean apparent prevalence ranged between states, Amhara 8.17 %, Benishangul Gumuz 13.86 %, Oromia 6.34 % and SNNPR 7.91 %. An overview of the identified survey districts with disease prevalence data is provided as Additional file 1.

### Meta-analysis and meta-regression

**Fig. 3:**
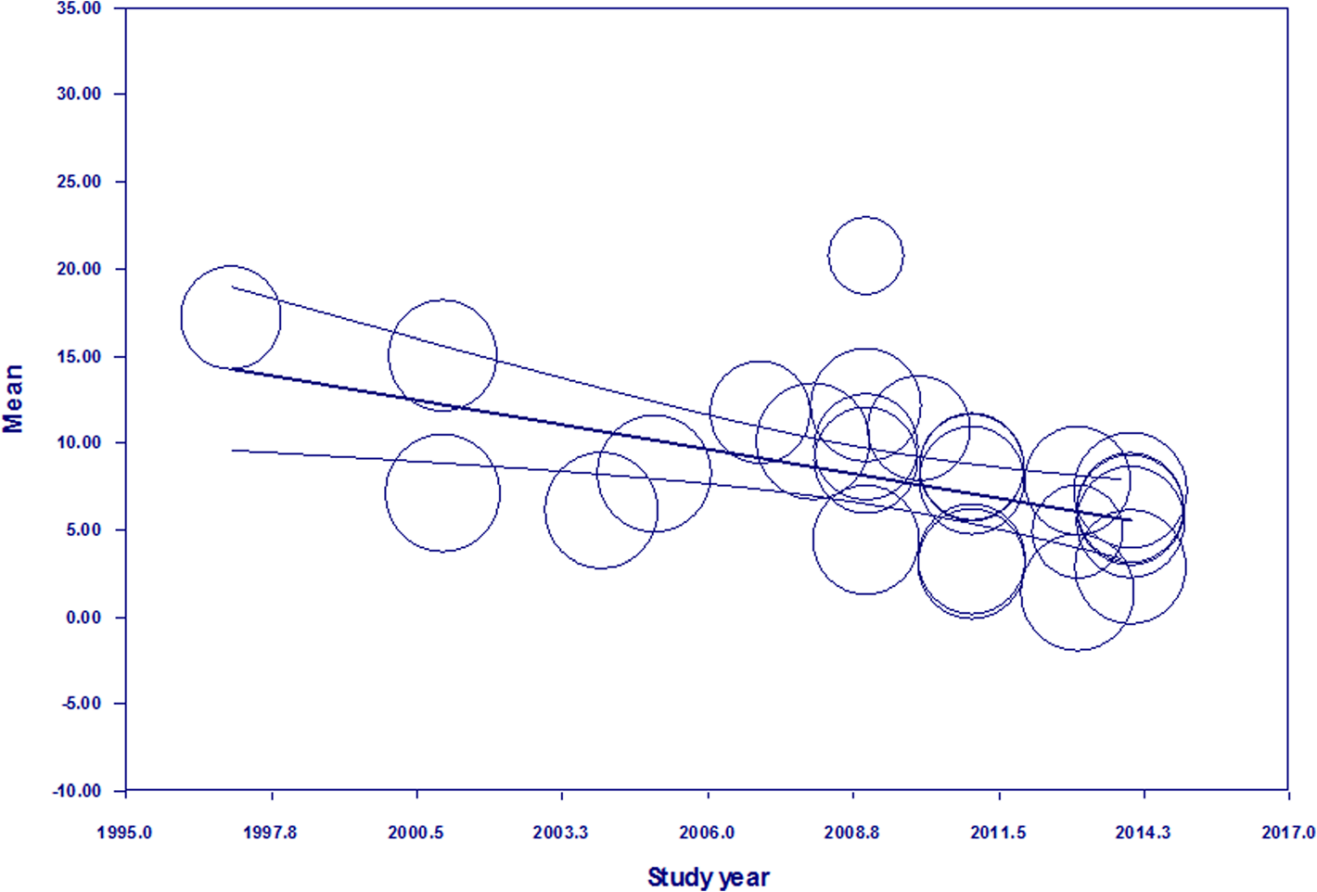
Regression of mean prevalence by year of study

Pooled prevalence estimates for the 24 studies included in the meta-analysis are presented in Table 2. Substantial heterogeneity was observed in the pooled estimate, which remained even after sub-group analysis. The pooled estimate of bovine trypanosomosis across studies for the entire period was 8.12 % (95 % CI: 6.88–9.35), ranging from 10.27 % (95 % CI: 7.34–13.20) in the late 1990s and early 2000s, to 6.81 % (95 % CI: 5.00–8.62) after 2010. Factors that could explain some of this variance were tested in the subsequent meta-regression. Results with coefficients, *p* values and Cochran’s Q Statistics from the univariable meta-regression analyses are outlined in Table 3. Region and survey year had a univariable *p* value <0.05, indicating a time-space stable pattern. A meta-regression of bovine trypanosomosis by survey year (consider here as a continuous variable) indicated a significant reduction in the prevalence of bovine trypanosomosis (*b* = −0.51; 95 % CI = −0.80; −0.23; *p* < 0.05). The scatterplot of regression of mean prevalence on year of study is presented in Fig. 3. Sub-group analysis by region revealed wide variations in prevalence. The highest estimated regional prevalence was 13.30 % (95 % CI: 7.73–18.88) in Benishangul Gumuz Regional state (Table 2) and was chosen as reference in the meta-regression. Compared to Benishangul Gumuz Regional State, Amhara, Oromia and SNNPR had significantly lower prevalence (*p* < 0.05) as shown in Table 3. Region, survey year and sample size explained 18, 5.5 and 2.7 % of the between study variance, respectively.

**Table 2:** Pooled prevalence estimates of bovine trypanosomosis, stratified by sub-groups

Characteristics	Number of studies	Pooled bovine trypanosomosis prevalence				Heterogeneity	
		Sample size	Cases	Prevalence (%)	95 % CI	*I* ^2^ %	*P*-value (Cochran’s Q)
Overall prevalence	24	27,719	2274	8.12	6.88–9.35	93.1	0.000
Region							
Afar	1	82	4	4.88	0.19–9.57	0.00	1.00
Amhara	6	15,937	1263	8.05	6.79–9.31	85.09	0.000
Benishangul Gumuz	4	2284	286	13.30	7.73–18.88	93.56	0.000
Oromia	8	2943	189	6.18	3.63–8.72	91.02	0.000
SNNPR	6	4045	366	7.87	4.56–11.19	92.93	0.000
Tigray	1	411	32	7.79	5.19–10.39	0.00	1.00
Survey year							
1997–2004	5	10,984	897	10.27	7.34–13.20	95.17	0.000
2005–2009	6	8059	1045	9.17	7.41–10.93	81.85	0.000
2010–2015	13	8676	332	6.81	5.00–8.62	92.18	0.000
Sample size							
< 384	3	959	84	8.25	3.99–12.51	84.01	0.002
384–1000	8	5982	482	8.72	7.16–10.29	93.96	0.000
≥ 1000	13	20,778	1708	7.74	5.49–9.98	92.78	0.000

**Table 3:** Regression coefficients, *p* values and Cochran’s Q statistics from univariate meta-regression of bovine trypanosomosis in Ethiopia

Variable	Category	Number	Coefficient	*p* value	Cochran’s Q statistics
Region	Benishangul Gumuz	2284	Reference		Q = 11.58, df = 5, *p* = 0.041
	Afar	82	−7.67	0.056	
	Amhara	15,937	−4.42	0.029	
	Oromia	2943	−6.45	0.001	
	SNNPR	4045	−4.70	0.022	
	Tigray	411	−4.76	0.172	
Survey year	1997–2004	10,984	Reference		Q = 7.33, df = 2, *p* = 0.026
	2005–2009	8059	−1.28	0.432	
	2010–2015	8676	−4.13	0.013	
Sample size	<384	959	Reference		Q = 0.75, df = 2, *p* = 0.686
	384–< 1000	5982	−0.59	0.774	
	>1000	20,778	0.57	0.786

### Publication bias

**Fig. 4:**
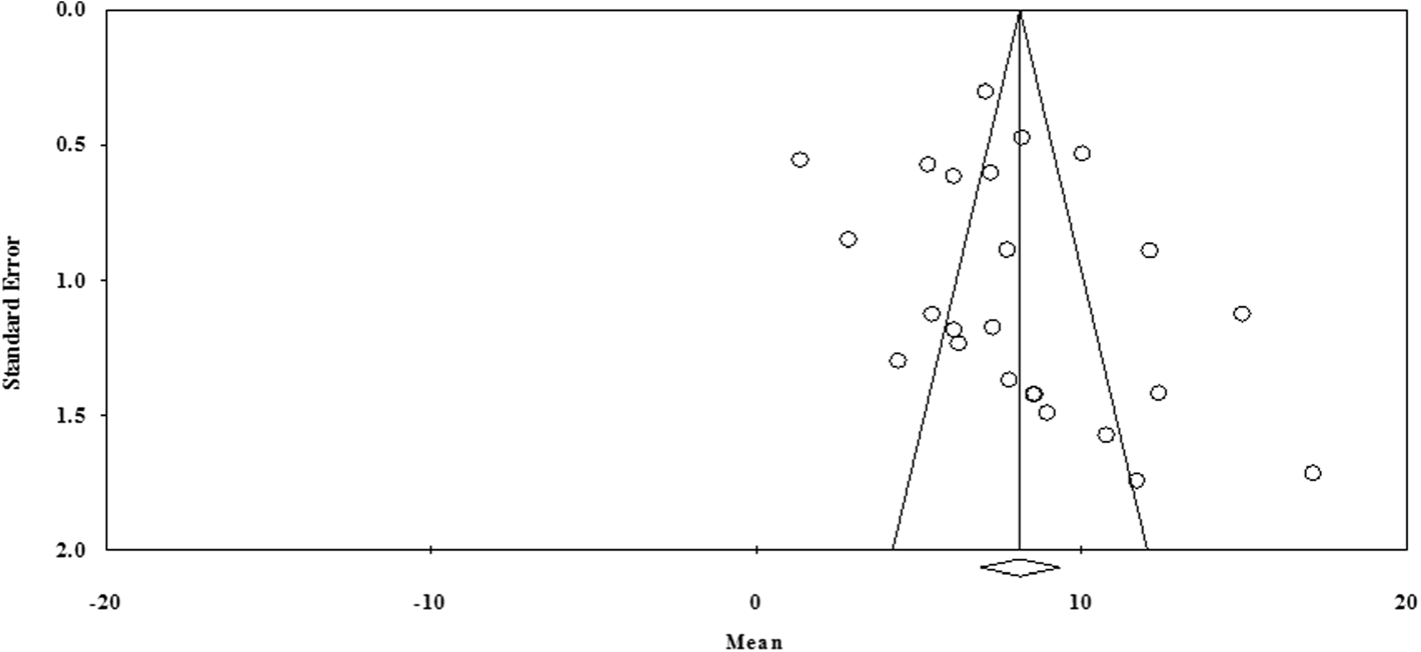
Funnel plots of the standard error by mean prevalence estimates

The funnel plots (Fig. 4) and the Egger’s regression asymmetry coefficient [*b* = 2.18] (95 % CI = −1.09, 5.46; *p* > 0.05) did not suggest the presence of publication bias, and no theoretical missing study was incorporated by the Duval and Tweedie’strim and fill method.

## Discussion

This study presents the first systematic nationwide analysis of bovine trypanosomosis prevalence in Ethiopia. It describes the prevalence estimates in Ethiopia derived from scientific reports. Bovine trypanosomosis prevalence varied over time, with lowest levels since 2010. Previous and on-going tsetse control activities in the affected areas of Ethiopia could be the reason for the decrement in the prevalence of bovine trypanosomosis. Significant variations have been observed in the prevalence of bovine trypanosomosis among regions. The variation between states would seem to indicate differences in effort and attitudes to tsetse and trypanosomosis control and eradication. The prevalence is significantly higher in Benishangul Gumuz Regional state, which is considerable remote with little tsetse control effort. The Ethiopian government, Pan-African Tsetse Eradication Campaign (PATEC) and International Atomic Energy Agency (IAEA) have been making a considerable effort to suppress tsetse fly challenge especially in Oromia, Amhara and SNNPR regions. Deltamethrin impregnated targets and Deltamethrin pour-on formulation have been widely used in Ethiopia to reduce tsetse challenge and the impact of trypanosomosis. However, In SNNPR, the Ethiopian government in association with IAEA have been using the sterile insect technique (SIT) to eradicate tsetse and reports indicate a significant reduction in tsetse and trypanosomosis challenge. The joint project between Ethiopian government and IAEA, Southern Tsetse Eradication Project (STEP), aimed to suppress tsetse from an estimated 25,000 km^2^. According to Gechere et al. [40], the fly density, trypanosomosis prevalence and mortality due to trypanosomosis have been significantly reduced in the project area. Additionally, the government of Ethiopia has conducted a massive settlement program in early 2000s to the tsetse and trypanosomosis belt. So, the destruction of the tsetse habitat by the settlers believed to lend a hand to the tsetse and trypanosomosis control programs in reducing the potential tsetse suitable areas.

The distribution of bovine trypanosomosis is found to be widespread covering most parts of the western and south-western parts of the country. This area is long known to be the major tsetse and trypanosomosis belt in Ethiopia [41, 42]. The area is one of the wettest and agriculturally productive parts of the country. Estimates made decades ago reported that 180,000–220,000 km^2^ land in the western and south western parts of the country to be suitable for tsetse, the biological vector of trypanosomosis. A recent estimate made by Leta et al. [43] reported that 140,000 km^2^ of fertile agricultural land which is roughly 12 % of the country’s landmass is found to be a suitable habitat for tsetse. However, it is important to note that the comparison between previous and present estimates should be done very carefully since different methodology had been applied. So, the difference in estimates observed may or may not reflect the decreasing risk of trypanosomosis in Ethiopia [43].

The pooled prevalence estimate varies significantly between regions. High prevalence of bovine trypanosomosis was reported from Benishangul Gumuz regional state. The studies were conducted mainly in endemic areas for bovine trypanosomosis and non-endemic regions and remote regions may possibly be under-represented. Even if the disease is known to be endemic to the Gambella Regional State, no study was reported from this region. Thus, the regional differences of data availability may have led to an over-estimation of the estimates for some regions. Due to the heterogeneous distribution of bovine trypanosomosis in Ethiopia, additional studies which are based on geostatistical techniques are needed. The studies must take into consideration distribution of tsetse and other biting flies, environmental factors, reservoir hosts, the control efforts made and settlement patterns.

Haematocrit centrifugation technique (HCT) was used in all the studies considered for the meta-analysis. Parasitological techniques like the HCT have been reported to be of low sensitivity but good specificity [44]. These techniques are of limited significance especially when parasitaemias are low as often observed in endemic areas [45]. According to Moti et al. [46], the percentage of positivity for the presence of trypanosomes detected by HCT was increased by more than 50 and 250 % when using PCR-RFLP-fp and PCR-RFLP-pb, respectively. This confirms the considerable under-estimation of the prevalence in research results reported and used in this analysis. Therefore, the pooled prevalence of bovine trypanosomosis reported in the study might be significantly lower than the real situation.

The study has some limitations. First, data showed a large degree of heterogeneity among studies, which remained even after sub-group analysis. Therefore, the findings may not necessarily reflect the real situation of the entire country. Other limitations are a consequence of incomplete or inaccurate information provided in the publications. Despite a comprehensive search, it is likely that some studies conducted have not been found because they are not published in journals indexed by PubMed. Even if the test for publication bias is not significant, it is difficult to exclude it from the limitations. In addition, studies were conducted between 1997 and 2014. This long time period was necessary because of the limited availability of data, but limits interpretation to some degree.

In Ethiopia, *T. congolense* is considered to be the most important trypanosome species. In this study *T. congolense* and *T. vivax* were found to be the two most predominant trypanosome species. The burden of non tsetse transmitted trypanosomosis like trypanosomosis caused by *T.vivax* is being reported to be considerable [24]. According to Fikru et al. [24], the occurrence of mechanically transmitted trypanosomosis caused by *T. vivax* is very widespread than previously thought. The high prevalence of *T. vivax* most likely indicates the local transmission in the non-tsetse infested area by biting flies. It is indicated that *T. vivax* can adapt to a non-tsetse dependent transmission cycle [47]. As a consequence, the eradication of tsetse fly in Ethiopia, which is a core focus area of Pan African Tsetse and Trypanosomosis Eradication Campaign and other tsetse control initiatives, might have little effect on the prevalence of *T. vivax*.

## Conclusion

To our knowledge, this is the first quantitative review attempt made on bovine trypanosomosis. The pooled bovine trypanosomosis prevalence estimate is high; however, higher degree of variability was observed between regions as well as survey year. The prevalence of the disease significantly reduces along time. The prevalence might be much higher if the studies used in the meta-analysis used highly sensitive molecular techniques. To this end, the use of molecular techniques for the diagnosis of bovine trypanosomosis should be adopted. *T. vivax* is reported to be one of the most important trypanosome species involved. Therefore, apart from the tsetse eradication campaign which is currently ongoing, efforts should also be made to control the mechanical transmission by biting flies such as tabanids, stomoxys and other potential mechanical vectors.

## Additional file

Additional file 1: PRISMA Checklist. (XLSX 25 kb)
